# Myocarditis Following COVID-19 Vaccination: A Systematic Review

**DOI:** 10.7759/cureus.37999

**Published:** 2023-04-22

**Authors:** Nour Shaheen, Abdelraouf Ramadan, Ahmed Shaheen, Mohamed Elmasry, Sarya Swed, Wael Hafez, Muhannad Wael

**Affiliations:** 1 Faculty of Medicine, Alexandria University, Alexandria, EGY; 2 Faculty of Medicine, Cairo University, Cairo, EGY; 3 Medicine, Aleppo University, Aleppo, SYR; 4 Internal Medicine, NMC (New Medical Centre) Royal Hospital, Abu Dhabi, ARE; 5 Internal Medicine, The National Research Centre, Cairo, EGY; 6 Faculty of Medicine, An-Najah National University, Jerusalem, PSE

**Keywords:** echocardiogram, electrocardiography, cardiac mri, myocarditis, sars-cov-2, covid-19 vaccines, covid-19

## Abstract

COVID-19 vaccination has significantly reduced both the morbidity and mortality rates associated with SARS-CoV-2 infection. Vaccines, especially mRNA vaccines, have been proposed in several studies to complicate viral myocarditis. Thus, our systematic and meta-analysis review aims to further investigate the possibility of an association between COVID-19 vaccines and myocarditis. We systematically searched PubMed, Web of Science, Scopus, Ovid, and Google Scholar and did a gray search of other databases using the following keywords and terms: "Myocarditis ("Myocarditis" Mesh) OR "Chagas Cardiomyopathy" Mesh) AND "COVID-19 Vaccines" Mesh. The studies were limited to only English articles that reported myocardial inflammation or myocarditis associated with COVID-19 vaccines. Pooled risk ratio with its 95% confidence interval was analyzed by RevMan software (5.4) to perform the meta-analysis. Our study included 671 patients from 44 studies with a mean age of 14-40 years. Nevertheless, myocarditis was noted in a mean of (3.227) days, and 4.19 per million vaccination recipients experienced myocarditis. Most cases were clinically presented with manifestations of cough, chest pain, and fever. Laboratory tests revealed increased C-reactive protein, and troponin with all other cardiac markers in most patients. Cardiac magnetic resonance imaging (MRI) revealed late gadolinium enhancement with myocardial edema and cardiomegaly. Also, electrocardiograms revealed ST-segment elevation in most patients. Furthermore, the incidence of myocarditis was statistically significantly lower in the COVID-19 vaccine group as compared with the control group (RR = 0.15, 95% CI = 0.10-0.23, p-value < 0.00001). No significant association was found between COVID-19 vaccines and the incidence of myocarditis. The study's findings highlight the importance of implementing evidence-based COVID-19 prevention strategies, such as vaccination, to reduce the public health impact of COVID-19 and its associated complications.

## Introduction and background

To the extent that the COVID-19 vaccination strategy advances, the effectiveness in other social groups and with a greater number of immunized patients will be documented. However, the effectiveness will be conditioned by the evolution of the pandemic itself, as well as other factors that are emerging, such as variants and the need for new vaccines or immunization boosters, which increase uncertainty about the best vaccination strategy to follow. Vaccine safety, or what is the same, the appearance of side effects of vaccines, is the second cornerstone, along with efficacy/effectiveness, which supports the strategy of any vaccine program and its acceptance by the population through the one that is directed. Undesirable post-vaccination reactions may be the result of an individual reaction of the vaccinated person to the administration of the vaccine, an implementation error or an administration error, or independent phenomena that occur concomitantly after vaccination without a causal relationship [1].

Myocarditis is an inflammatory disease of the heart muscle (myocardium), with a wide range of clinical presentations, etiologies, and therapeutic responses [1]. There are many potential causes of myocarditis including viruses, including the ones that cause the common cold (adenovirus); coronavirus disease 2019 (COVID-19); bacteria; parasites; and fungi. Myocarditis may result from exposure to a long list of medicines or illegal drugs that cause allergic or toxic reactions, such as chemotherapy, antibiotics such as penicillin and sulfonamide, and some anti-seizure medications [2].

SARS-CoV-2, a novel species of coronavirus, announced the beginning of the COVID-19 pandemic on March 11, 2020 [3]. As of December 2020, vaccination campaigns have begun in different countries. There have been several vaccine candidates tested and found to be safe and effective against COVID-19. COVID-19 vaccines were developed and utilized relatively quickly compared to other vaccines. As a result, monitoring their efficacy, safety, and side effects requires continuous and extensive research [4].

The United States Food and Drug Administration (FDA) approved the emergency use authorizations (EUAs) for the Pfizer-BioNTech mRNA vaccine (BNT162b2) and for the Moderna mRNA vaccine (mRNA-1273) in December 2020 [5]. Both of the initial studies to establish the efficacy of mRNA vaccines did not report any cases of myocarditis [6,7].

Serious adverse events have recently been reported with COVID-19 vaccines, especially RNA (mRNA) vaccines [8,9]. In April 2021, Israel reported the first cases of myocarditis following vaccination with mRNA [10]. Furthermore, on June 20, 2021, the CDC declared myocarditis and pericarditis as side effects of the COVID-19 vaccine [11,12]. There are several molecular mechanisms for vaccination-related myocarditis: cardiovascular tissues include a large number of angiotensin-converting enzyme 2 (ACE2) receptors, which the SARS-CoV-2 spike protein binds to. Viral antigens or other proteins secreted by injured cardiomyocytes can activate naive T lymphocytes, causing inflammation. As previously primed T cells target both the spike protein in the vaccine and the cardiac antigens, a prior COVID-19 infection may further increase the risk of myocarditis after immunization. Another potential mechanism involves molecular mimicry between the self-antigens and the spike protein [12].

In this systematic review and meta-analysis, we are going to evaluate the clinical features, diagnostic tests, and incidence of myocarditis after receiving COVID-19 vaccines.

## Review

Methods

Protocol and Registration

The systematic review was conducted following the Preferred Reporting Items for Systematic Reviews and Meta-Analyses (PRISMA) checklist. However, the protocol was not registered [12].

Study Search and Inclusion Criteria

The inclusion criteria for the published studies included the following: 

Case reports of myocarditis following mRNA COVID-19 vaccine administration but tested negative for COVID-19 and provided a detailed account of the ensuing medical treatment.

Exclusion Criteria

The exclusion criteria for the studies include the following:

1) Articles that are not case reports, case series, or randomized control trials. 2) Articles that were reviews or editorials. 3) Studies in languages other than English.

Information Sources and Search Strategies

In the present study, a systematic literature review was performed via PubMed, Scopus, Web of Science, Ovid, Embase, and Google Scholar to investigate the predictive role or diagnostic value of electrocardiogram (ECG) in patients with COVID-19 infection. For this purpose, "Myocarditis" and "COVID-19 vaccine" with all their equivalents and different written forms as key terms were searched in the PubMed as follows: (("Myocarditis"Mesh) OR "Chagas Cardiomyopathy"Mesh) AND "COVID-19 Vaccines"Mesh for PubMed and other databases TITLE-ABS-KEY (("Myocarditis" AND (covid-19 AND vaccine*)). The synthesis of data was conducted without strict inclusion criteria in order to minimize the risks of bias.

First, we searched only English articles, and then we excluded review articles, book chapters, and conference papers. Our PICO (Population, Intervention, Comparison and Outcomes) was P: patients receiving any of COVID-19 vaccines, I: COVID-19 vaccines, C: control, O: myocarditis.

Study Selection

Inclusion criteria were assessed by examining titles or abstracts. Full-text articles were then read, and any that did not satisfy the inclusion criteria or met the exclusion criteria were excluded.

Data Collection Process and Data Items

Articles related to myocarditis were extracted for information such as the name of the first author, the year and country of publication, and the design of the study. Study participants' age, gender, type of vaccine, PCR test, and presenting complaints were collected from all studies. Cohort studies provided data also on study participants' age, gender, type of vaccine, and PCR test along with the control group that did not take the vaccine.

Risk of Bias Within the Studies

The study characteristics were used to analyze potential biases in the included studies. The papers were assessed by two independent reviewers (A.R., A.S.) for their methodological quality. A third reviewer (N.S.) assessed papers for which a conclusion could not be reached. In this systematic review, we used the Joanna Briggs Institute's critical appraisal tool for case reports and cohort studies [13]. A checklist of eight questions in the Appendix was used to determine whether bias existed for each article. Based on the bias scores, the articles were classified into three categories: low (included), high (excluded), and uncertain (more information is needed). According to the checklist, if the answer to half or more of the eight questions on it is "yes," then the study is at low risk of bias; similarly, if the answer is "no," it is at high risk of bias. In contrast, "unclear" answers had a response rate greater or equal to 50%.

Results

Study Selection

**Figure 1:**
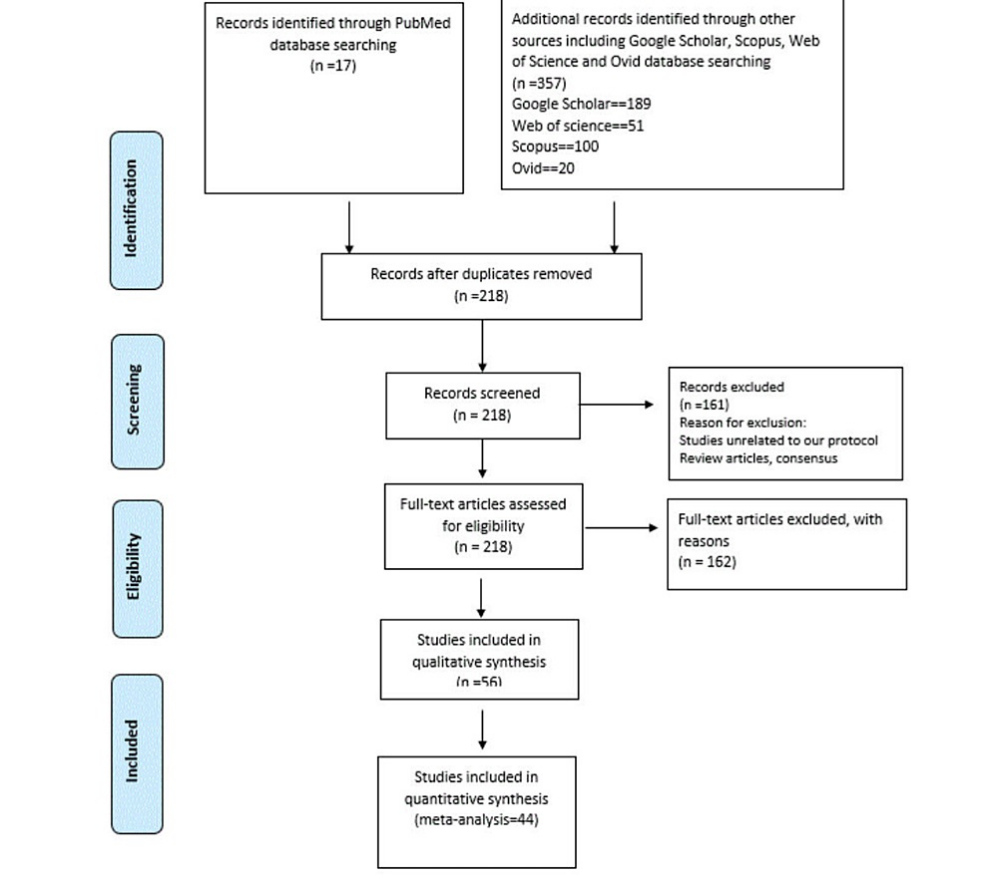
Prisma flow diagram of the study screening and inclusion

We searched six databases (PubMed, Scopus, Web of Science, Ovid, Embase, and Google Scholar) for articles about myocarditis after COVID-19 vaccines. A total of 44 related articles were found out of 357 after excluding duplicates and irrelevant articles; three cohorts were eligible for the meta-analysis. Twenty-six were case reports, 14 were case series, and one was a letter included in the systematic review (PRISMA flow diagram) in Figure 1, showing the Prisma flow diagram of the study screening and inclusion.

Based on our systematic review of 44 studies, we identified 26 case reports, 14 series and one letter as well as three cohort studies. In these studies, 203 cases came from the USA, 17 from Israel, two from Italy, one from Korea, one from Poland, two from Qatar, and one from Spain, and 471 cases came from multi-country cohort studies.

There were 462 males and 209 females. One hundred and five had myocarditis, 13 acute myocarditis, 19 peri myocarditis, and four fulminant myocarditis. Among the patients with myocarditis, the mean age was 27.8 ± 13.7 years, and the most common vaccine was Pfizer-BioNTech with 192 cases, followed by Moderna with 93 cases, Pfizer and Moderna with six cases, and Johnson & Johnson with three cases. The results of reverse transcription polymerase chain reaction (RT-PCR) testing in 146 patients were negative and the results for COVID-19 in 22 patients were positive as shown in Table 1.

**Table 1:** Demographic and general information of the included studies

Reference	Country	Study type	No. of patients	Mean age	Male/female (gender)	Possible diagnosis	Type of vaccine	PCR test
Ammirati et al. [1]	USA	Case report	2	21.25	1 male, 1 female	Myocarditis	Moderna	Negative
Jain et al. [14]	USA	Retrospective multicenter	63	15.6	58 males, 5 females	Acute myocarditis	59 Pfizer-BioNTech, 4 Moderna	38 negative, 18 positive, 10 not tested
Li et al. [15]	Multi-country	Cohort	471	NA	190 females, 281 males	Myocarditis pericarditis	NA	NA
Hasnie et al. [17]	USA	Case report	1	22	1 male	Peri myocarditis	Moderna	Negative
Albert et al. [18]	USA	Case report	1	24	1 male	Myocarditis	Moderna	Negative
Habib et al. [19]	Qatar	Case report	1	37	1 male	Myocarditis	Pfizer-BioNTech	Negative
Kim et al. [20]	Korea	Case report	1	24	1 male	Acute myocarditis	Pfizer-BioNTech	Negative
Verma et al. [21]	USA	Case report	2	43.5	1 female, 1 male	Fulminant myocarditis	Pfizer-BioNTech	Negative
García et al. [22]	Spain	Case report	1	39	1 male	The definitive etiological diagnosis was difficult to determine	Pfizer-BioNTech	Negative
Khogali and Abdelrahman [23]	Qatar	Case report	1	29	1 female	Pericarditis	Moderna	Negative
Snapiri et al. [24]	Israel	Case report	7	16.8	7 males	Peri myocarditis	Pfizer-BioNTech	Negative
Larson et al. [25]	USA	Case series	8	31.6	8 males	Myocarditis	2 Moderna, 6 Pfizer-BioNTech	Negative
Abbate et al. [26]	USA	Case report	2	27 (died), 34	1 male, 1 female	1 patient died due to recurrent cardiac arrest and refractory shock fulminant myocarditis	Pfizer-BioNTech	Negative
Das et al. [27]	USA	Case series	25	15.2	22 males, 3 females	Myopericarditis	Pfizer	COVID 19 testing done for 15 out of 25. Four positives
Schauer et al. [28]	USA	Case series	13	15	12 males, 1 female	Myopericarditis	Pfizer	Negative
Sokolska et al. [29]	Poland	Case report	1	21	1 male	The vaccine-associated acute myocarditis was diagnosed	Pfizer-BioNTech	Negative
Muthukumar et al. [30]	USA	Case report	1	52	1 male	Myocarditis	Moderna	Negative
Park et al. [31]	USA	Case report	2	15.16	2 males	Self-limited myocarditis	Pfizer-BioNTech	Negative
Deb et al. [32]	USA	Case report	1	63	1 male	Acute myocarditis	Moderna	Negative
Levin et al. [33]	Israel	Case series	7	20.4	7 males	Myocarditis	Pfizer-BioNTech	Negative
Minocha et al. [34]	USA	Case report	1	17	1 male	Acute myocarditis	Pfizer-BioNTech	Negative
Nassar et al. [35]	USA	Case report	1	70	1 female	Myocarditis	Janssen	Negative
Watkins et al. [36]	USA	Case report	1	20	1 male	Myocarditis	Pfizer-BioNTech	Negative
Marshall et al. [37]	USA	Case series	7	16.7	7 males	All patients in this series had myocarditis or myopericarditis	Pfizer-BioNTech	Negative
Abu Mouch et al. [38]	Israel	Case series	6	16-45, median 22	1 male	Myocarditis	Pfizer-BioNTech	Negative
Starekova et al. [39]	USA	Case series	5	17-38	4 males, 1 females	Myocarditis	3 Pfizer-BioNTech, 2 Moderna	Negative
Dionne et al. [40]	USA	Case series	15	12-18	14 males, 1 female	Myocarditis	Pfizer-BioNTech	Negative
Isaak et al. [41]	USA	Case report	1	15	1 male	Vaccine-induced hypersensitivity myocarditis	Pfizer-BioNTech	Not available
Montgomery et al. [42]	USA	Case series	23	20-51	1 male	Myocarditis	7 Pfizer-BioNTech, 16 Moderna	19 negative, 4 not performed
Rosner et al. [43]	USA	Case series	7	24	1 male	Myocarditis	1 Johnson & Johnson, 1 Moderna, 5 Pfizer-BioNTech	Negative
Cereda et al. [44]	Italy	Case report	1	21	1 male	Acute myocarditis	Pfizer-BioNTech	Negative
McLean and Johnson [45]	USA	Case report	1	16	1 male	Myopericarditis	Pfizer-BioNTech	Negative
Nevet [46]	Israel	Case series	3	24.3	1 male	Acute myocarditis	Pfizer-BioNTech	Not available
King et al. [47]	USA	Case series	4	25.5	3 males, 1 female	Myocarditis	3 Moderna, 1 Pfizer Bio-NTech	3 negative, 1 unknown
Dickey et al. [48]	USA	Case series	6	27	1 male	Myocarditis	Pfizer-BioNTech and Moderna	Negative
Patrignani et al. [49]	Italy	Case report	1	56	1 male	Acute myocarditis	Pfizer-BioNTech	Negative
Sulemankhil et al. [50]	USA	Case report	1	33	1 male	Myocarditis	Janssen	Not available
Araja D [51]	USA	Case report	1	24	1 male	Acute myocarditis	Pfizer-BioNTech	Negative
Hudson et al. [52]	USA	Case report	2	22.24	1 male	Myopericarditis	Pfizer-BioNTech	Negative
Tano et al. [53]	USA	Case series	8	16	8 males	Perimyocarditis	Pfizer-BioNTech	Negative

Cohort Studies

Three cohort studies were included in the meta-analysis, 546 patients had myocarditis out of 130,158,303 of the vaccinated group, and 24 patients developed myocarditis out of 884,828 of the non-vaccinated control group. We found a statistically significant association between the decreased incidence of myocarditis and receiving COVID-19 vaccines compared with the control group (RR = 0.15, 95% CI = 0.10-0.23, p < 0.00001). This statement is made by analyzing all three cohorts collectively. The incidence of myocarditis was 4.19 per 1 million people who received the vaccines.

In the first retrospective cohort study [14], there were 63 adolescent patients with myocarditis following COVID-19 vaccination (58 males and five females), with a mean age of 15.6 years. Fifty-nine patients (94%) had received the Pfizer-BioNTech vaccine and four (6%) had received the Moderna vaccine. All except for one patient presented following the second dose. In the real-time reverse transcription polymerase chain reaction (RT-PCR) test, 38 were negative, 18 were positive, and 10 were not tested. Six percent of patients had significant dysrhythmia, and 14% had mild left ventricular dysfunction on echocardiography which resolved on discharge. Eighty-eight percent met the diagnostic cardiac magnetic resonance (CMR) Lake Louise criteria for myocarditis. At a mean of 35 days on following up data, 86% of patients showed resolution of symptoms, arrhythmias, and ventricular dysfunction.

In the second retrospective study [15], 471 (190 females and 281 males) out of 126,661,070 persons in a large multinational retrospective cohort study characterizing adverse events after COVID-19 vaccines, which included eight medical records and five administrative claims databases from Australia, France, Germany, Japan, Netherlands, Spain, the United Kingdom and the United States, developed myocarditis pericarditis.

In the third retrospective study [15,16], the Pfizer-BioNTech vaccine was associated with an excess risk of myocarditis (1 to 5 events) per 100,000 persons mainly after the second. The median age of the 21 persons with myocarditis in the vaccinated group was 25 years and 90.9% of the patients were male. A summary of findings from all studies is presented in Table 1.

Synthesis of Results (Case Reports and Case Series)

In terms of time to symptoms appearing, it was six days mainly after the first dose and three days after the second dose of the vaccine. For patients who had the first dose, the hospitalization time was four days, and for those who had the second dose, it was three days (Table 1).

Presenting Complaints

On presentation, 93 cases were represented with chest pain as a predominant complaint, 34 of them suffered from fever, 33 suffered from dyspnea, and two had a persistent cough, as shown in Table 2.

**Table 2:** Clinical manifestations among patients with myocarditis after COVID-19 vaccines on admission

Symptom	References	Total (N)
Chest pain	[17,20,23,25,26,29,30,32-38,44,46,47,50-53]	93
Fever	[18-23,25,26,32,34,37,38,41]	34
Dyspnea	[20,21,25,32,35,37,38,40,44,51,52]	33
Cough	[25,26]	2

Laboratory Values

The mean temperature recorded was elevated in some patients and decreased in others. C-reactive protein (CRP) levels were generally increased. Moreover, in all studies, troponin-I or troponin-T levels were increased along with creatinine kinase-myocardial band (CK-MB) levels and brain natriuretic peptides (BNP) (Table 3).

**Table 3:** Laboratory values of patients with myocarditis after COVID-19 vaccines WBC, white blood cell; CRP, C-reactive protein; CK-MB, creatinine kinase-myocardial band; BNP, brain natriuretic peptide.

Vitals and lab values	Trends	References	(Standard range)
Vitals			
Temperature, Celsius	Elevated	[5,17,37,38,51]	(<37.5)
Temperature, Celsius	Decreased	[5,30,36,38]	(>37.5)
Systolic blood pressure, mmHg	Increased	[5,30,36,38]	(90-120)
	Normal	[5,17,35,38,49,51]	
Diastolic blood pressure, mmHg	Increased	[33,38]	(60-80)
	Decreased	[17,36,38,51]	
	Normal	[5,30,35,38,49]	
Arterial blood gas			
pCO_2_, mmHg	High	[32]	(35-45)
pO_2_, mmHg	Normal	[35]	(75-100)
Inflammatory markers			
WBC, cells/mm^3^	Elevated	[19,30,37,52]	(4,500-11,000)
CRP, mg/L	Elevated	[18,20,25,26,37,38,40,41,43,44,47,49,50-53]	(<8.0)
Cardiac markers			
Troponin-I, ng/mL	Elevated	[5,18-21,25,27-29,32,33,35,37,38,42-44,47-49,50,51,53]	(<0.04)
Troponin-T, ng/mL	Elevated	[5,18-21,25,27-31,35,37,38,40-42,47-53]	(<0.01)
CK-MB, ng/mL	Elevated	[18,20,26,30,44,51,53]	(<5.0)
BNP, pg/mL	Elevated	[28,29,32,38,40,51,52]	<125

Common Findings on Diagnostic Tests

The diagnostic tests and imaging techniques used in the study are listed in Table 4. All patients tested positive for COVID-19. A bilateral infiltrate was found in two chest radiographs, and bilateral ground glass opacities were seen in all chest computed tomography (CT) scans [18,21]. Heart magnetic resonance imaging (MRI) revealed late gadolinium enhancement in 40 patients, and 41 images revealed myocardial edema. Imaging studies revealed cardiomegaly or vascular redistribution in six patients. The CT angiography studies did not detect coronary artery stenosis except in one case.

**Table 4:** Common findings on diagnostic tests of patients with myocarditis after COVID-19 vaccines

Tests	Total\frequency (N)	Proportion recorded from articles reviewed
	Imaging (X-ray, computed tomography [CT], magnetic resonance imaging [MRI])	
Bilateral infiltrates/ground glass opacities	2	[18,21]
Myocardial edema	41	[5,17,19-23,25,26,29,32,33,36,37,41,43,47,49,50,51,53]
Late gadolinium enhancement	40	[5,18,25,26,33,37,38,40,41,43,46,49,50,53]
	Echocardiography	
Decreased left ventricular ejection fraction (LVEF) (55%-70%)	15	[25,32,35,36,38,43,51]
Cardiomegaly or wall thickness	5	[5,19,32,33,49]
Vascular redistribution	1	[36]
Coronary artery stenosis	1	[48]
	Electrocardiography	
ST-segment elevation	21	[5,17,19,20,25,29,32,33,36,38,40,41,43,44,46-49,53]
T-wave inversion	1	[20]

The electrocardiograms showed ST-segment elevation in 86 cases, and inverted T waves in two cases. In seven studies, two-dimensional (2-D) echocardiography revealed decreased left ventricular ejection fraction (LVEF), and in five cases, cardiomegaly or increased wall thickness was observed (Table 4).

Discussion

To the best of our knowledge, our systematic review and meta-analysis are the most updated to further investigate the incidence of myocarditis in COVID-19-vaccinated patients. After receiving COVID-19 vaccines, the most prevalent clinical manifestations are fever, cough, and fatigue [54]. Myocarditis typically presents with fever, dyspnea, and/or chest pain, which makes it difficult to recognize and diagnose during the vaccination. In this systematic review, we identified that patients with COVID-19 myocarditis commonly experience dyspnea, coughing, fever, and chest pain.

In conventional medicine, serum biomarkers are used to diagnose suspected acute myocarditis. Troponin was elevated in a few patients with acute myocarditis [55]. In fulminant myocarditis, serum cardiac troponins are almost always elevated [56,57]. Many patients have been reported to have elevated troponin levels after receiving COVID-19 vaccines, with particular differences observed between deceased and surviving patients [57]. Myocarditis, however, cannot be ruled out in the absence of increased serum cardiac troponins [58].

The levels of CK-MB and BNP are often elevated in myocarditis and may provide insight into prognosis [59]. CRP was commonly elevated, though normal levels did not exclude myocarditis. CRP was also above the normal range in most patients [58].

A wide range of ECG abnormalities including ST-segment abnormalities were observed in most patients with myocarditis. The ECG of most patients with myocarditis demonstrated non-specific characteristics such as sinus tachycardia, ST-wave and T-wave abnormalities, and occasionally an atrioventricular or bundle branch block [58]. Even though ST-segment elevation in contiguous leads in a segmental or nonvascular pattern indicates myocarditis, they are often misinterpreted as coronary occlusions [60].

In histologically proven myocarditis, echocardiographic patterns consistent with dilated, hypertrophic, restrictive, and ischemic cardiomyopathies have been described, as well as increased left ventricular sphericity and volume [61]. In this systematic review, almost all myocarditis patients had cardiomegaly, pleural effusion, and decreased LVEF.

As a result of active inflammation, dilated ventricles are characteristic of acute myocarditis, though fulminant myocarditis can also result in increased left ventricular wall thickness. Many of the cases reviewed here were associated with cardiomegaly [61].

This systematic review also found that some patients developed pneumonia as evidenced by ground glass opacities on chest CTs [18,21].

Management of Myocarditis

Seven studies used corticosteroids in the treatment procedures [20,25,33,37,38,46,52], while human immunoglobulin was used in each of the seven studies as a single therapeutic intervention [21,26-28,34,38]. The utilization of nonsteroidal anti-inflammatory drug (NSAID) was described in 10 studies [20,24,25,28,37,38,52].

Outcome

Most studies include patient outcomes. Two deaths were reported, and it was related to myocarditis [27,53]. All other articles describe either a complete or partial recovery.

Limitations of the Study

Statistical analyses were not conducted since there were no control or comparison groups in case reports and case series studies but were carried out in cohort studies. Additionally, the second multinational cohort, which included more than 100 million people, is at risk of bias. Since the study included a follow-up of one year or more, there is a possibility that there was an underreporting of the number of myocarditis patients.

## Conclusions

Myocarditis induced by COVID-19 vaccination is not reported as a significant association. International cohort studies are needed to clarify the actual occurrence, identify the physiological mechanism, and determine the best strategic approach due to the seriousness of the condition.

## Appendices

**Table 5:** Summary assessment of the risk of bias for the included studies

Study ID	Year	Were the patient's demographic characteristics clearly described? (yes/no)	Was the patient's history clearly described and presented as a timeline? (yes/no)	Was the patient's clinical condition on presentation clearly described? (yes\no)	Were diagnostic tests or assessment methods and results clearly described? (yes\no)	Was the intervention or treatment procedure(s) clearly described? (yes\no)	Was the post- intervention clinical condition clearly described? (yes\no)	Were adverse events (harms) or unanticipated events identified and described? (yes/no)	Does the case report provide takeaway lessons? (yes/no)	Total score
Ammirati et al. [1]	2021	No	No	Yes	Yes	Yes	Yes	No	Yes	62.5%
Jain et al. [14]	2021	Yes	No	Yes	Yes	Yes	Yes	Yes	Yes	87.5%
Li et al. [15]	2021	No	No	No	No	No	No	No	Yes	12.5%
Hasnie et al. [17]	2021	Yes	Yes	Yes	Yes	Yes	Yes	Yes	Yes	100%
Albert et al. [18]	2021	Yes	No	Yes	Yes	No	No	Yes	Yes	62.5%
Habib et al. [19]	2021	Yes	Yes	Yes	Yes	Yes	Yes	Yes	Yes	100%
Kim et al. [20]	2021	No	Yes	Yes	Yes	No	No	Yes	Yes	62.5%
Verma et al. [21]	2021	No	No	Yes	No	No	No	No	Yes	25%
García et al. [22]	2021	Yes	Yes	Yes	Yes	No	No	No	Yes	62.5%
Khogali et al. [23]	2021	No	Yes	Yes	Yes	Yes	Yes	Yes	Yes	87.5%
Snapiri et al. [24]	2021	No	No	No	Yes	Yes	Yes	Yes	Yes	62.5%
Larson et al. [25]	2021	Yes	No	Yes	Yes	No	Yes	No	Yes	62.5%
Abbate et al. [26]	2021	Yes	Yes	Yes	Yes	Yes	Yes	Yes	Yes	100%
Sokolska et al. [29]	2021	No	Yes	Yes	Yes	No	No	No	Yes	50%
Muthukumar et al. [30]	2021	Yes	Yes	Yes	Yes	Yes	Yes	Yes	Yes	100%
Park et al. [31]	2021	Yes	No	Yes	Yes	Yes	Yes	Yes	Yes	87.5%
Deb et al. [32]	2021	No	No	Yes	Yes	Yes	Yes	Yes	Yes	75%
Levin et al. [33]	2021	Yes	No	No	No	No	No	No	Yes	25%
Minocha et al. [34]	2021	No	Yes	Yes	Yes	Yes	Yes	Yes	Yes	87.5%
Nassar et al. [35]	2021	No	No	Yes	Yes	Yes	Yes	Yes	Yes	75%
Watkins et al. [36]	2021	No	No	Yes	Yes	Yes	No	No	Yes	50%
Marshall et al. [37]	2021	No	No	Yes	Yes	Yes	Yes	Yes	Yes	75%
Abu Mouch et al. [38]	2021	No	No	Yes	Yes	Yes	No	No	Yes	50%
Starekova et al. [39]	2021	No	No	No	Yes	No	No	No	Yes	25%
Dionne et al. [40]	2021	No	No	Yes	Yes	Yes	Yes	No	Yes	62.5%
Isaak et al. [41]	2021	No	No	Yes	No	No	Yes	Yes	Yes	50%
Montgomery et al. [42]	2021	No	No	Yes	Yes	Yes	No	No	Yes	50%
Rosner et al. [43]	2021	No	No	Yes	Yes	Yes	No	No	Yes	50%
Cereda et al. [44]	2021	No	Yes	Yes	Yes	No	No	No	Yes	50%
McLean and Johnson [45]	2021	Yes	Yes	Yes	Yes	Yes	Yes	Yes	Yes	100%
Nevet [46]	2021	No	No	No	No	Yes	No	No	Yes	25%
King et al. [47]	2021	No	No	Yes	No	Yes	Yes	Yes	Yes	62.5
Dickey et al. [48]	2021	No	No	No	No	No	No	No	Yes	12.5%
Patrignani et al. [49]	2021	No	No	Yes	Yes	Yes	Yes	Yes	Yes	75%
Sulemankhil et al. [50]	2021	No	Yes	Yes	Yes	No	Yes	No	Yes	62.5%
Araja D [51]	2022	Yes	Yes	Yes	Yes	Yes	Yes	Yes	Yes	100%
Hudson et al. [52]	2021	No	No	Yes	No	Yes	Yes	No	Yes	50%
Tano et al. [53]	2021	No	No	Yes	Yes	Yes	Yes	No	Yes	62.5%
